# A Systematic Study of Restorative Crown-Materials Combinations for Dental Implants: Characterization of Mechanical Properties under Dynamic Loads

**DOI:** 10.3390/ijms23158769

**Published:** 2022-08-07

**Authors:** Xavier Marimon, Miguel Cerrolaza, Miquel Ferrer, Oriol Cantó-Navés, Josep Cabratosa-Termes, Román Pérez

**Affiliations:** 1 Bioengineering Institute of Technology, Universitat Internacional de Catalunya (UIC), 08195 Barcelona, Spain; 2 Automatic Control Department, Universitat Politècnica de Catalunya (UPC-BarcelonaTECH), 08028 Barcelona, Spain; 3 Department of Strength of Materials and Structural Engineering, Universitat Politècnica de Catalunya (UPC-BarcelonaTECH), 08028 Barcelona, Spain; 4 School of Dentistry, Universitat Internacional de Catalunya (UIC), 08195 Barcelona, Spain

**Keywords:** FEA, dental implants, dynamical forces, biomechanical behavior, restorative materials, crown materials

## Abstract

This study aimed to find the optimum mechanical characteristics of the restorative materials for the manufacture of implant crowns subjected to impact loading when different combinations of materials are used for the inner and outer crown. Several combinations of external–internal crown restorative materials were analyzed. The dynamic stresses at eight different zones of a dental implant subjected to an impact load and the influence of several mechanical properties, such as the Young’s modulus, Poisson’s ratio, density, and initial velocity, were analyzed and compared. A detailed 3D model was created, including the crown, the retention screw, the implant, and a mandible section. The model was then built by importing the 3D geometries from CAD software. The whole 3D model was carefully created in order to guarantee a finite element mesh that produced results adjusted to physical reality. Then, we conducted a numerical simulation using the finite element method (FEM). The results of the FEM analysis allowed for evaluating the effect that different combinations of restorative materials and mechanical properties had on the stress distribution in various regions of the implant. The choice of restorative material is a factor to be considered in order to preserve the integrity of osseointegration. Restorative materials transfer more or less stress to the dental implant and surrounding bone, depending on their stiffness. Therefore, an inadequate Young’s modulus of the rehabilitation material can affect the survival of the implant over time. Eight interactive graphics were provided on a web-based surface platform to help clinical dentists, researchers, and manufacturers to select the best restorative materials combination for the crown.

## 1. Introduction

Nowadays, clinical evidence of higher fracture risk and biological issues has been observed depending on the materials used to manufacture dental implant crowns (ceramics, composites, metals, etc.). The main biological problem that dental implants can present is peri-implantitis, especially in patients with a higher risk of gingival inflammation, such as smokers and/or those with poor hygiene, previous periodontitis, or genetic factors [1,2,3,4,5].

The stresses generated in the chewing process seem to be at the origin of this problem [6,7]. Many simulations and experimental tests have studied this issue from a static approach, applying constant predefined forces or pressures onto the implant crowns [8,9,10]. Consequently, the stress values and distributions on the implant body were observed to be independent of the used crown material. Indeed, according to Saint Venant’s principle, the difference between the effect of several statically equivalent loads applied to a system becomes insignificant at a sufficiently large distance from the loading area.

In fact, the predefinition of such static force intensity hides a crucial mechanical aspect: the real dynamic chewing forces acting in the crowns depend on the whole system stiffness in a real dynamic scenario, especially the crown material stiffness itself. Therefore, it is necessary to conduct a dynamic simulation to approach the effect of the crown material on the mechanical behavior of the implant.

The material choice for implant-supported crowns is a determining factor for the survival of prostheses and implants [11,12], since the crown stiffness plays a crucial role during the collision since the maximum force and maximum stress depend on how it absorbs the impact energy [13,14,15,16,17,18,19,20,21,22]. In this project, an in-depth study of the mechanical response was conducted for different Young’s modulus of the crown materials, density, Poisson’s ratio, and initial impact velocity. The maximum stresses over time are presented through a 3D response surface, depending on the mentioned variables.

The main objective of this research is to find the ideal mechanical properties of restorative materials for the manufacture of implant crowns under impact loading. Different Young’s moduli have been combined for both outer and inner crowns. Three-dimensional graphs have been created to predict the behavior of the implant with any crown materials by knowing their Young’s moduli only. The density of the materials has been adjusted as a function of Young’s modulus.

## 2. Results and Discussion

The dynamic behavior of the implant and the surrounding bone is of the most concern when studying impact loads that produce a transient response. Therefore, this section is devoted to analyzing and comparing the dynamic results focusing on two main aspects: (a) the dynamic evolution of the stress distribution, and (b) a graphical comparison to evaluate the influence of the Young’s modulus on the stresses distribution at all the selected nodes of the model. The model was created with Solidworks R2022 [23] using as a reference a 3D scan of the implant. The simulation has been performed with Ansys v2022 R2 [24].

### 2.1. Influence on Stress Distribution

**Figure 1:**
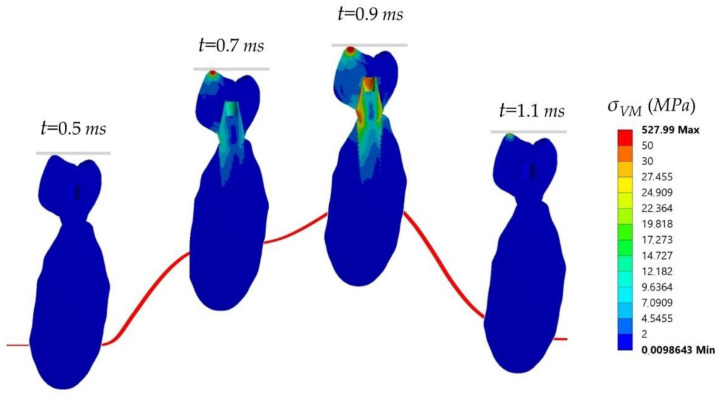
Distribution of Von Mises stresses (MPa) on the longitudinal section of model carbon fiber ceramic crown (FCCER) after the impact at different time steps. From left to right: 0.5 ms, 0.7 ms, 0.9 ms, and 1.1 ms. Adapted from [21].

The stress distribution varied over time, reaching the maximum stress values during the impact. Due to the impact eccentricity, the crown and the implant started bending around the *z*-axis, and some oscillations occurred. As mentioned previously, there was a singular point at the highest crown cusp; therefore, the values obtained at that zone were not representative. Figure 1 shows the stress evolution at the significant time instants in the cross-section of one of the carbon fiber ceramic crown (FCCER) model.

**Figure 2:**
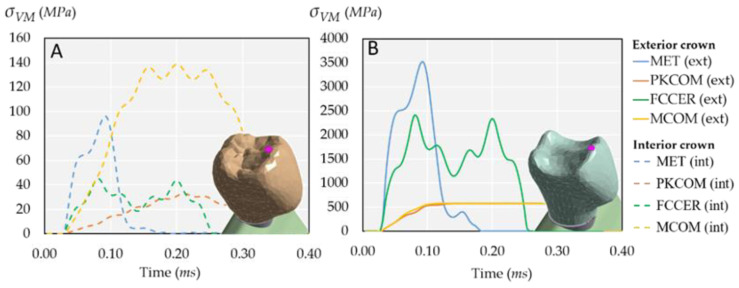
Von Mises stress at the selected nodes in both internal and external parts of the crown for the common materials: MET, PKCOM, FCCER, and MCOM. (**A**) Interior crown. (**B**) Exterior crown.

It can be seen that the stress was higher at the impact zone, and it was transferred then through the crown, thus concentrating on the abutment where the crown rests. The stress distribution did not significantly vary among the different cases. The maximum stress occurred at the crown’s top (528 MPa), whereas in the abutment, the values were about 40 MPa and around 0.5 MPa at the apex. Figure 2 shows comparisons of the Von Mises stresses at the two parts of the crown for the previously studied materials.

### 2.2. Stress Response over Time

**Figure 3:**
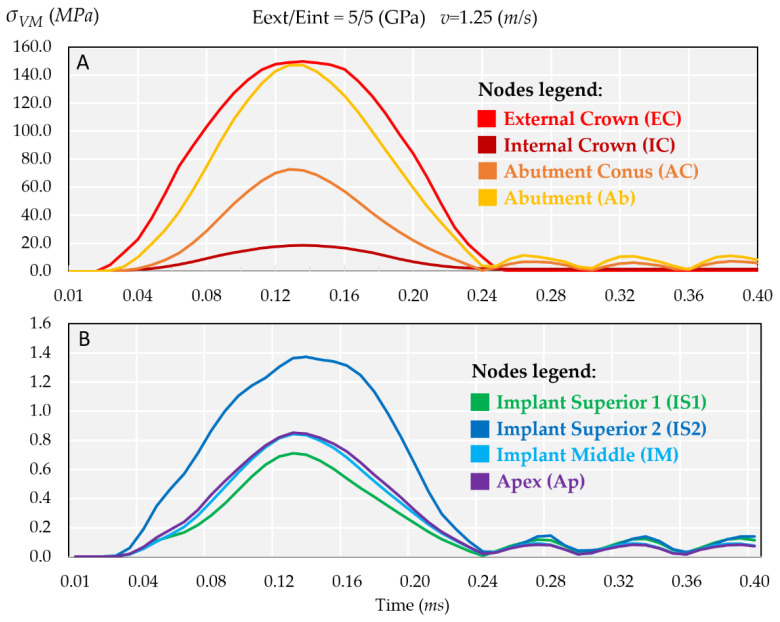
Comparison of the dynamic behavior of Von Mises stress, see Figure 4 for nodes’ locations. (**A**) Four superior nodes. Note the significant difference in stress between the external crown (EC) and the internal crown (IC). (**B**) Four inferior nodes. Note the difference in stress between the apex (Ap) and the implant superior 2 (IS2) nodes.

**Figure 4:**
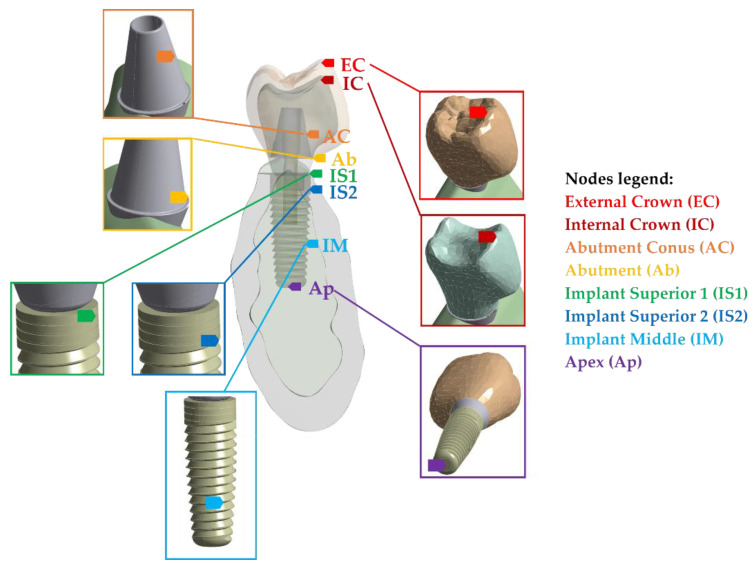
Reference nodes used for numerical simulation.

Figure 3A,B shows the evolution over time of Von Mises stress at all the selected nodes of the model (see Figure 4).

The stress evolution was displayed in two figures because of the large difference in stress values, ranging from 150 MPa to 0.7 MPa. As expected, the highest stresses were at the external crown (EC), 150 MPa, while the lower stresses were registered at the internal crown (IC) as 20 MPa, as shown in Figure 3A. Regarding the lower four nodes, there was a large difference between IS1 and Ap nodes (Figure 3B) because the impact force was gradually balanced along the implant length from IS2 node until the end of the implant.

### 2.3. Influence of Young’s Modulus on External and Internal Crowns

The results obtained from the dynamic analysis are presented through maximum stress surfaces at each node. This graphic aims to rapidly evaluate the better combination of materials and estimate the magnitude of internal stresses generated by a material combination.

**Figure 5:**
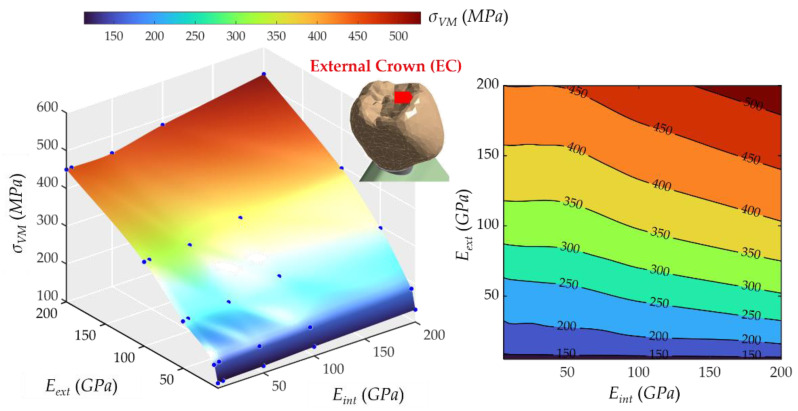
Von Mises maximum stress at the external crown (EC). (**Left**): 3D representation. (**Right**): vertical projection.

Figure 5 shows that the external crown behavior was similar to the apex; although it was more proportional to the *E_ext_*, the stress ranged from 113.39 MPa to 527.21 MPa. Indeed, the higher the stiffness, the higher the stress response, following a linear law. Moreover, the effect of the *E_int_* was negligible. The maximum value also occurred in the case of the most rigid crowns.

**Figure 6:**
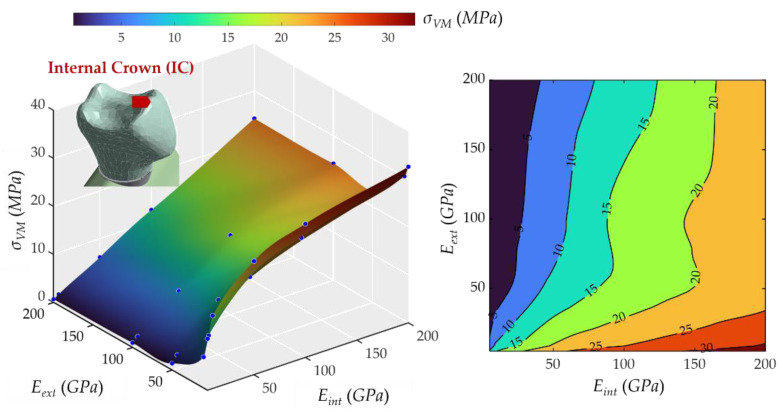
Von Mises maximum stress at the internal part of the crown, internal crown (IC). (**Left**): 3D representation. (**Right**): vertical projection.

Figure 6 shows that the *E_int_* influence on stresses at the internal part of the crown was also proportional. However, the E*_ext_* slightly affected the stress, contrary to the *E_int_*, so the more rigid the external crown, the lower stress at the internal crown. The stress varied from 0.67 to 32.52 MPa, and the peak occurred at the combination of the most rigid internal part of the crown and the softest external part of the crown. Additionally, it can be seen that a remarkable effect occurred in the range from 0 to 50 GPa of the external crown.

**Figure 7:**
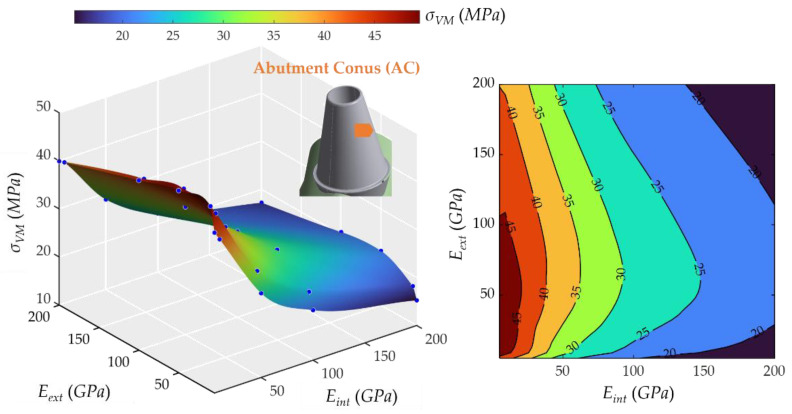
Von Mises maximum stress in the conic surface of the abutment, abutment cone (AC). (**Left**): 3D representation. (**Right**): vertical projection.

As for the abutment cone, Figure 7 shows that the less rigid the internal part of the crown, the higher the stress at the conic surface of the abutment. The behavior was nonlinear with the rigidity of the external crown since the highest stress values occurred when the *E_ext_* was 50 MPa. The lowest value was 20.42 MPa, and the highest was 48.42 MPa. The effect of the *E_ext_* crown was very small, while the *E_int_* had a more significant influence on the stress evolution.

**Figure 8:**
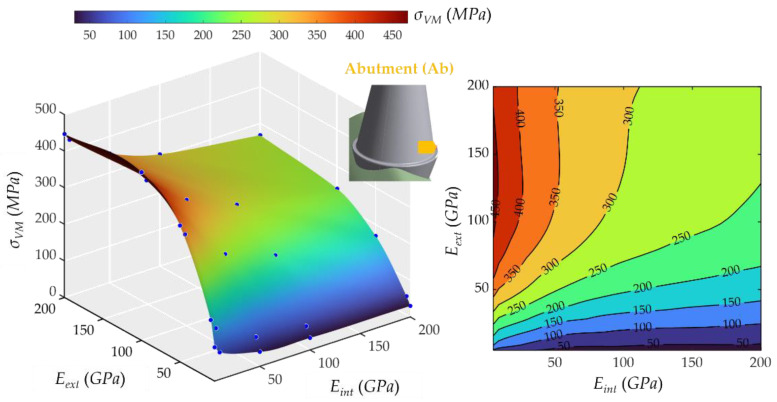
Von Mises maximum stress at the middle part of the abutment (Ab). (**Left**): 3D representation. (**Right**): vertical projection.

The base of the abutment behaved differently, as shown in Figure 8. *E_ext_* was determining more the stress on this part, unlike the case above. Moreover, the *E_int_* had a slight decreasing effect on stresses. The highest stress was 459.77 MPa, and the lowest was 30.25 Mpa. The *E_ext_* had a decisive influence on stresses, and for large values of the *E_ext_*, the *E_int_* began to have appreciable effects; a decrease was observed in *σ_VM_*, approaching a constant value around 300 MPa, usually known as a “plateau” zone, i.e., a flat region.

**Figure 9:**
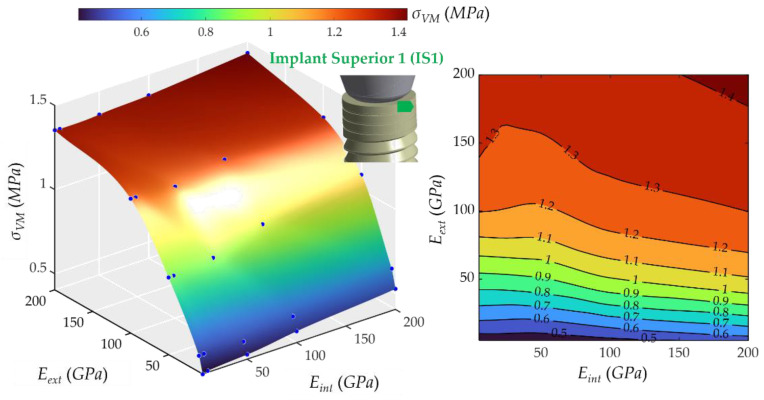
Von Mises maximum stress at the first upper part of the implant, implant superior 1 (IS1). (**Left**): 3D representation. (**Right**): vertical projection.

The behavior was remarkably similar to the apex stress response regarding the first upper part of the implant (see Figure 9). The *E_ext_* had a steady increasing effect on the stress, whereas the *E_int_* did not have almost any effect. The stress varied from 0.40 to 1.42 MPa.

**Figure 10:**
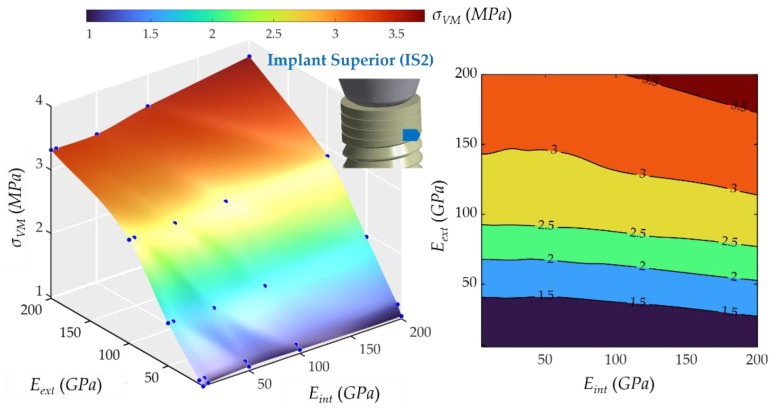
Von Mises maximum stress at the second upper part of the implant, implant superior 2 (IS2). (**Left**): 3D representation. (**Right**): vertical projection.

The same behavior was found in the second upper part of the implant (see Figure 10). The lowest stress was 0.97 MPa, and the highest was 3.72 MPa. The *E_ext_* had a decisive influence, and the *E_int_* practically did not affect the value of *σ_VM_*. However, the *σ_VM_* increased more than in the IS1 for similar values of *E_ext_*.

**Figure 11:**
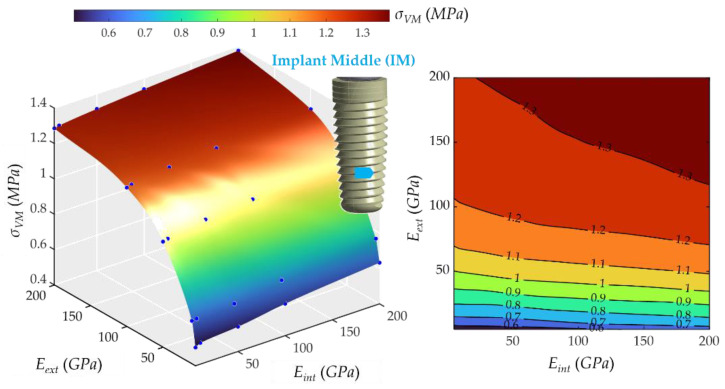
Von Mises maximum stress at the middle part of the implant. (**Left**): 3D representation. **(Right**): vertical projection.

Likewise, the middle part of the implant (see Figure 11) behaved similarly. The lowest stress was 0.50 MPa, and the highest was 1.37 MPa. The behavior of *σ_VM_* was very similar to that of implant superior 1 (IS1), with a decisive influence of *σ_VM_* and very little of *E_int_*.

**Figure 12:**
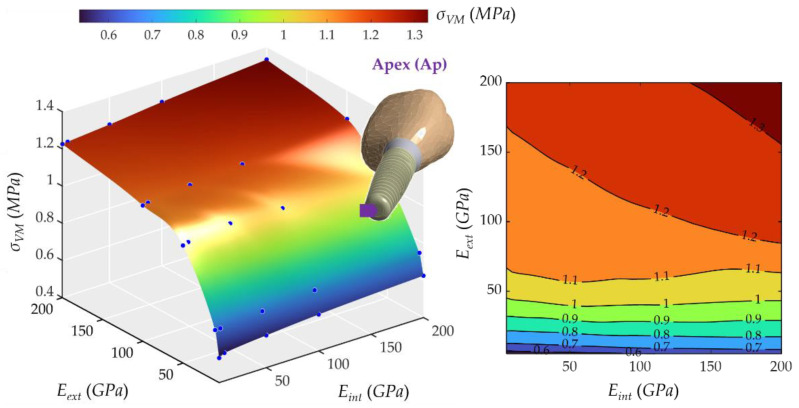
Von Mises maximum stress in the apex (Ap). (**Left**): 3D representation. (**Right**): vertical projection.

Figure 12 shows how the Young’s modulus of external and internal crowns affected the maximum stress in the apex. It can be noted that stresses increased steadily from 0.53 MPa to 1.33 MPa when increasing *E_ext_*. In contrast, *E_int_* did not significantly change the stress. The peak occurred in the case of the most rigid crowns. The behavior of *σ_VM_* was very similar to that of implant superior 1 (IS1) and implant middle (IM), with a decisive influence of *E_ext_* and very little of *E_int_*.

**Figure 13:**
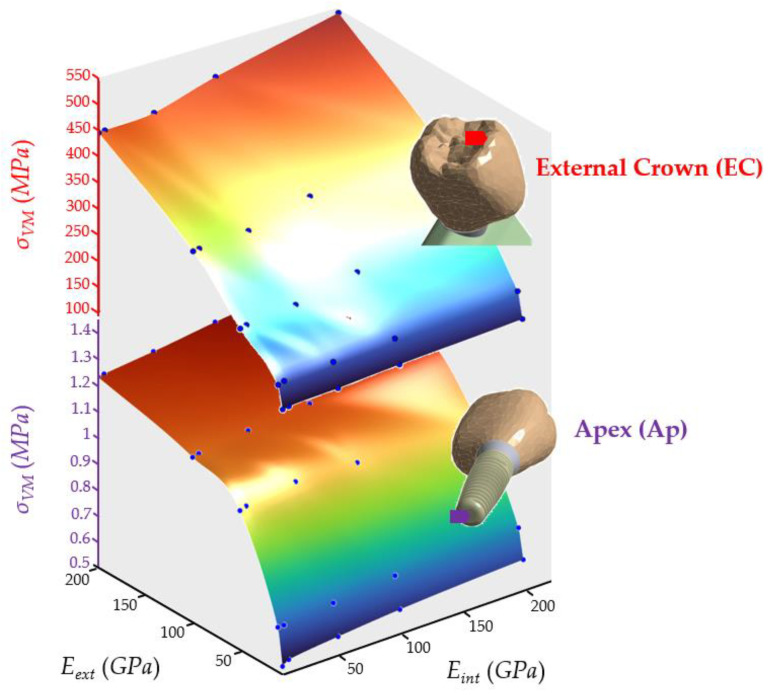
Comparison of Von Mises stresses between the external crown node (EC) and the apex node (Ap).

Von Mises maximum stress, *σ_VM_*, at the apex node (Ap) varied more slowly than at the external crown node (EC) due to mechanical damping effects of materials, including both trabecular and cortical bone. This effect is important in the dynamic response because it avoids oscillatory effects. It is critical for peri-implantitis because if Von Mises stress is small in the implant, especially in the Apex (Ap), this will reduce the risk of peri-implantitis, especially in patients with gingival inflammation (see Figure 13).

Regarding the real materials frequently used in dental implantology (MET, MCER, MCOM, FCOM, PKCOM, FCCER), the combinations, and their dynamic responses have already been studied, reported, and validated in previous works by the same authors [1,2,20]. The results of these simulations with the exact properties of the real materials matched very well within the stress surfaces (see Figure 5, Figure 6, Figure 7, Figure 8, Figure 9, Figure 10, Figure 11 and Figure 12).

## 3. Materials and Methods

### 3.1. The Implant–Bone Model

**Figure 14:**
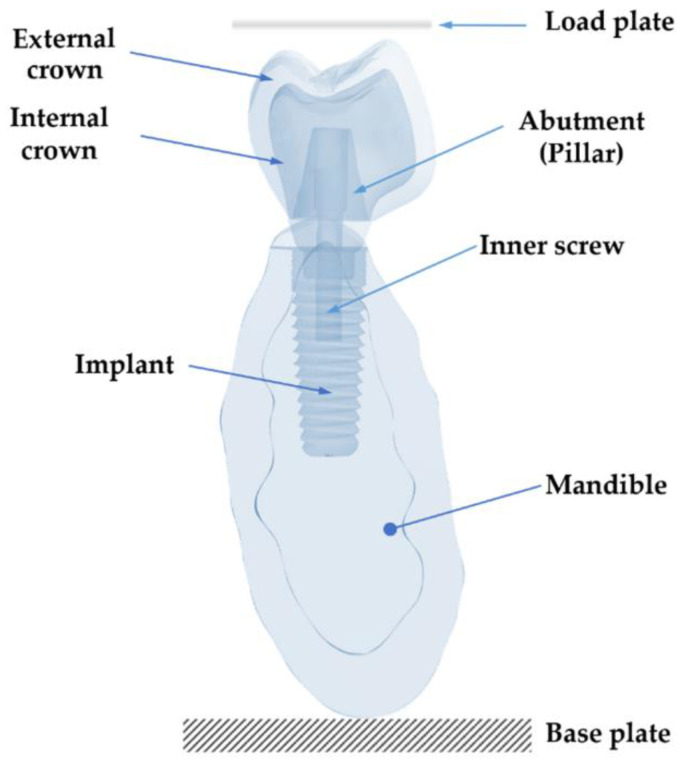
Longitudinal-section view of the whole 3D model: load plate, crowns, pillar, inner screw, implant, cortical and cancellous bones.

The endosseous implant used in this study is sized 4.2 × 11.5 mm [25]. The implant was modeled in Solidworks CAD software v2022 R2 [23]. The whole studied system consists of the following components: crowns, the inner screw, the abutment, the implant, and the load plate (see Figure 14). All the parts of the model are described hereafter. Ethics approval was not required for this in vitro study.

### 3.2. Load Plate

**Figure 15:**
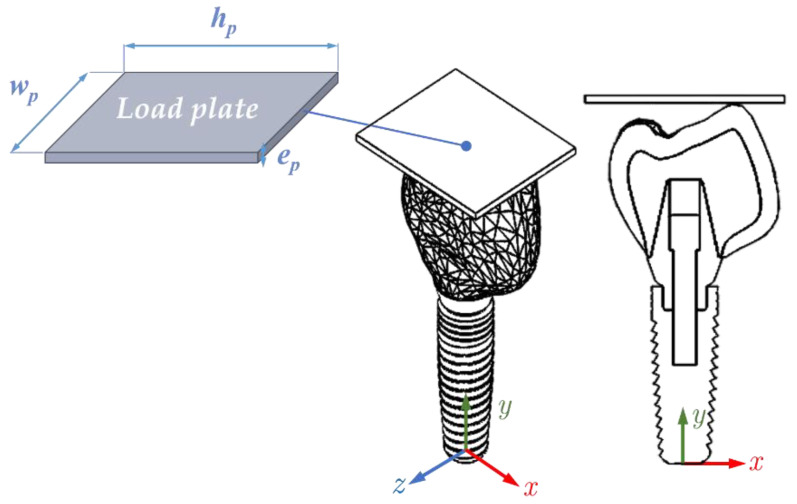
Whole implant and load plate (*w_p_* = 10, *h_p_* = 12, *e_p_* = 2 mm). For simplicity, the bone structure is not shown.

A fixed and flat rigid body was required to simulate the impact load on the tooth during masticatory function. A rectangular-shaped plate (*w_p_* = 10, *h_p_* = 12, *e_p_* = 2 mm, see Figure 15) was modeled to apply the impact load on the whole model. The initial distance between the plate and the crown is 0.01 mm, and the contact is assumed to be frictionless. The contact separates freely, with no resistance.

The load plate movement and deformation are completely restrained so that it acts as an ideal rigid surface and do not absorb any impact energy. Therefore, the implant elements below the plate are the only elements involved in the impact energy absorption.

The impact energy is given in terms of the velocity’s magnitude and is discussed in Section 3.4.

### 3.3. Materials of the Model

The crown can consist of two different materials: one for the inner part and another for the outer. In this study, the materials of these two parts will vary throughout the simulations. Some of the most common materials and combinations used for the inner and outer parts are listed in Table 1 below.

The mechanical properties of the referred crown materials are listed in Table 2 below.

This study carried out a a multiple-configuration finite-element analysis to explore different material combinations for inner and outer crown parts, with generic Young’s modulus ranging from 5 to 200 GPa and densities from 2 to 10 g/cm^3^. The Poisson’s ratio was set to 0.3 for all cases. The mechanical properties of the fictitious are listed in Table 3 below.

The results of these analyses, as discussed later on, will allow for the estimation of the critical stresses by knowing the combination of the crown materials.

Both the abutment and the implant were built with Ti-6Al-4v, an alloy that contains 6% aluminium and 4% vanadium (see Table 4). The load plate was modeled as 10 times stiffer than steel, aiming to have quasi-rigid plates. Two types of bone were considered: cortical bone and trabecular bone.

All materials were considered to be elastic, homogeneous, and isotropic. The mechanical properties of all these components are shown below:

It should be remarked that the mechanical properties of the materials, specifically their stiffness (*E*, *ν*) and mass (*ρ*), directly influence the dynamic response of the system, particularly on the maximum stresses and deformation.

### 3.4. Numerical Simulation

Although many studies focus on static forces, few studies on the real dynamic masticatory forces can be found. Nevertheless, some biomechanical estimated values can be obtained in the literature [12,13].

An impact between the rigid load plate and the implant–bone model was carried out with the dynamic inputs explained below. As corresponds to a transient phenomenon, the results were analyzed as time-dependent rather than stationary.

The simulation was performed with Ansys Workbench Software v2022 R2 [24] using the transient mechanical solver and Newmark’s implicit time-integration algorithm.

As the characteristics and severity of impacts depend on the kinetic energy and momentum involved, i.e., the masses and velocities, these two magnitudes must be unified in all the models so that tests can be compared.

#### 3.4.1. Finite Element Mesh

**Figure 16:**
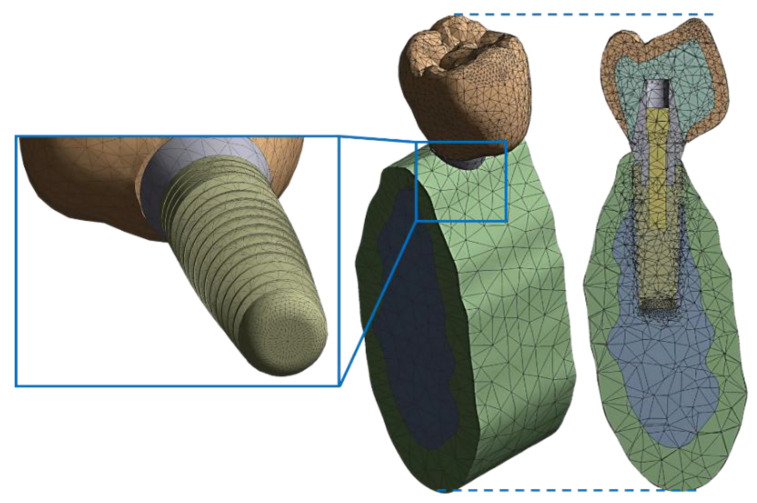
Finite element mesh. Left: detailed view of mesh at the apex. Center: perspective view. Right: cross-sectional view. Adapted from [20].

The results accuracy depends directly on the quality of the finite element mesh and the size of the elements. Medium mesh size with an adaptive size meshing algorithm was used. In addition, the mesh was refined in the contact area to ensure better accuracy in this zone. Figure 16 shows the resulting finite element mesh, built with 115,828 nodes and 77,419 tetrahedral elements.

On the other side, the size of the elements affects both the results accuracy and the computing time. The larger the element size, the larger the error and the shorter the computational time. While the computing time is not relevant in static analyses, it is crucial in transient dynamic studies.

#### 3.4.2. Boundary Conditions

In a dynamic analysis, instead of stationary forces set as boundary conditions, the impact conditions are set up in terms of initial displacements, velocities, or accelerations of an object. The simulation end-time also has to be set. The chewing impact occurs in a very short period of time. Some previous works in other scientific fields found that the impact lasts less than one millisecond [32]. On the one hand, the duration of the simulations should be as short as possible to reduce the CPU computation time; but on the other hand, it should be longer than the impact duration and its further effects. Finally, the simulation end-time was set at 0.4 ms, corresponding to the total duration of the transient. The number of substeps, or time-step, has to be fixed, where substeps is the number of halfway calculations to change from a geometry to the next one. A value of 53 substeps, i.e., a time-step of 7.55 µs, was found to be reasonable. Selected nodes are displayed and identified in Figure 4.

Two different stress values can be obtained from the IS1 node to the Ap node, as these four nodes are in the bone–implant interface: a higher stress value at the implant (as it is stiffer) and a lower stress value at the bone. The bone-stress value was used since the bone is the region of interest.

Extremely high stress arises when a load is applied to a tiny area. In our case, the impact occurred at a single point, in the highest dup of the crown. In reality, this is practically impossible because every collision always occurs between two surfaces, even if they are very small, not between two points. The result is that the stress tends to an infinite value around that point. For this reason, several nodes were selected close to the singular point, so that their values were more realistic. The mesh is the same for all the models, and then the selected nodes are also the same. As shown in Figure 4, the chosen node for the internal crown (IC) is located on the contact surface between the external and internal parts of the crown. The closest node to the impact zone is the node at the external crown (EC).

Two nodes were selected in the abutment: the abutment node (Ab) and the abutment cone node (AC). The former was located at the abutment base, while the latter was located at the conical region because stresses are rather homogenous in the conical part.

Four nodes were selected in the implant. All of them were in the interface between the screw and the bone mesh: the implant superior 1 (IS1) and implant superior 2 (IS2), the implant middle node (IM), and the apex node (Ap). Two stresses can be obtained at these points: one from the screw mesh and the other from the trabecular bone. As the screw and the bone stiffness are very different, this makes the stress response. It is important to note again that the studied stresses are the ones belonging to the bone.

### 3.5. Simulation Parameters

The following variables were set to be parameters in this study, ordered according to their relevance:

- External crown’s Young’s modulus (*E_ext_*). According to the mechanical properties listed in Table 1 and Table 2, the external crown Young’s modulus varied from 200 GPa (MET) to 8.43 GPa (MCOM, FCOM, and PKCOM) but was not equally spaced between the models. Then, discrete values for this parameter were set to 10, 50, 100, and 200 GPa.
- Internal crown’s Young’s modulus (*E_int_*). According to the mechanical properties listed in Table 1 and Table 2, the internal crown’s Young’s modulus varied from 200 GPa (MET) to 4.1 GPa (PKCOM) but was not equally spaced between the models. Then, discrete values for this parameter were set to 5, 10, 50, 100, and 200 GPa.
- Initial velocity (*v*). The velocity, together with the global mass of the system, directly defined the impact magnitude, i.e., the kinetic energy transferred to the dental implant. Consequently, the initial velocity was set uniformly for all the simulations, particularly *v* = 1.25 m/s.
- Density (*ρ*). As described above, the system mass, i.e., the material densities also affected the impact energy. First, it can be observed from the crown material models (see Table 2) that the higher the density of the crowns, the higher the Young’s modulus, although not proportionally. From this premise, a benchmark of densities values was defined for each Young’s modulus, identified as *ρ*_used_ (see Table 5). Then, two levels of density were defined as (+) and (−), each of one with higher and lower values of density, respectively. Therefore, the density parameter is discretized by establishing three different sets of density values that vary according to Young’s modulus. Table 5 collects the density values.
- Poisson’s ratio (ν). It was taken as 0.3 for all simulations.
- Friction. The collision with the plate was frictionless. This means that a null friction-coefficient was assumed that allowed for a free sliding. In addition, normal pressure was equal to zero if separation occurred.

### 3.6. Study Cases

**Figure 17:**
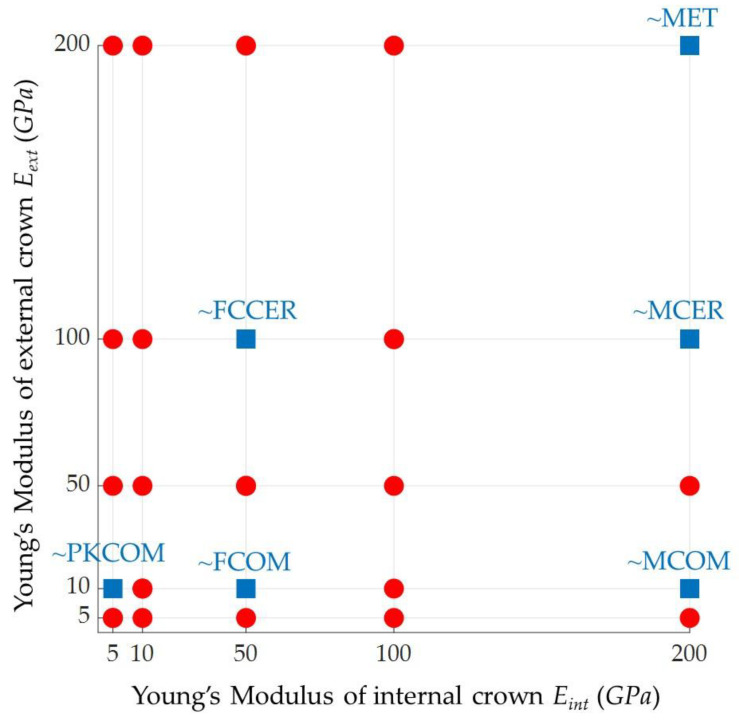
Combinations of Young’s modulus, *E*, in GPa for all study cases. Red balls: generic materials. Blue squares: studied cases with mechanical properties close to standard materials.

The study cases analyzed in this study are shown in Figure 17 below. There were 4 × 5 simulations for each combination of the Young’s modulus (GPa). The equivalents of the six crown material models are indicated. For each of these simulations, the reference values of parameters were *v* = 1.25 m/s, density *ρ*_used_ (see Table 5), and Poisson’s ratio *ν* = 0.3.

## 4. Conclusions

We found evidence using a numerical simulation about the influence of elastic parameters on the stress response on the different parts of the implant under a dynamic load. It can be said that the case that overall presents higher stress values is the extreme case of the highest Young’s modulus of both the external and internal parts of the crown, that is, the MET implant, the one that is made of only metal material.

Apart from the impact zone, the highest stresses always appear on the abutment and the bone–implant contact interface. This corner is also placed on the same side as the impact point, so it suffers the bending moment created by the impact excentricity. Therefore, the choice of material for implant-supported crowns is a factor to consider for the survival of prostheses and implants.

From the surface response plots of the maximum stress, it is evident that there was a direct correlation between the Young’s modulus of the crown materials and the stresses on almost all parts of the dental implant, following a similar pattern. It should be emphasized that this difference was remarkable at the crowns. However, its effect was not always the same. The relationship was linear in the apex, the external and internal parts of the crowns, and the implant, but, in the abutment base, the maximum stress occurred at the lowest values of *E_int_* and medium values of *E_ext_*.

Finally, eight interactive graphic surfaces (Figure 5, Figure 6, Figure 7, Figure 8, Figure 9, Figure 10 and Figure 11) are provided on a web-based platform to help in the selection of the best combination of restorative crown materials.

## Figures and Tables

**Table 1:** Common combination of materials for inner and outer crowns.

Inner Crown	Outer Crown	Combination ID
Cr-Co	Cr-Co	MET
Cr-Co	Ceramics VMK 95	MCER
Cr-Co	Composite BioXfill	MCOM
Fiber of BioCarbon	Composite BioXfill	FCOM
PEEK	Composite BioXfill	PKCOM
Fiber of BioCarbon	Ceramics IPS e.max	FCCER

**Table 2:** Mechanical properties of the crown materials.

Material	Young Modulus (GPa)	Density (g/cm^3^)	Poisson’s Ratio *ν*	Manufacturer or/and References
Cr-Co	200	10	0.31	[22]
Ceramics VMK 95	91	2.40	0.20	Vita [26]
PEEK	4.1	1.30	0.36	Invibio [27]
Composite BioXfill	8.43	2	0.20	Micro-Medica [28]
Fiber of BioCarbon	66	1.40	0.30	Micro-Medica [28]
Ceramics IPS e.max	95	2.50	0.20	Ivoclar Vivadent [29]

**Table 3:** Young’s moduli and densities explored in this study.

Generic Material	Young’s Modulus *E*(GPa)	Density *ρ* (g/cm^3^)	Poisson’s Ratio *ν*
Material 1	5	2	0.3
Material 2	10	2	0.3
Material 3	50	2	0.3
Material 4	100	5	0.3
Material 5	200	10	0.3

**Table 4:** Mechanical properties of the implant, bone and plates.

		Young’s Modulus *E* (GPa)	Density *ρ* (g/cm^3^)	Poisson’s Ratio *ν*	Manufacturer or/and References
Implant	Ti-6-Al-4V	113.8	4.43	0.34	MIS implants [25]
Bone	Cortical bone	15	1.79	0.3	[30,31]
	Trabecular bone	0.5	1.79	0.45	[31]
Plates	Load and base plates	2000	8	0.3	

**Table 5:** Benchmark of densities (*ρ*) as a function of Young’s modulus (*E*).

Young’s Modulus *E* (GPa)	Density *ρ*_used_ (g/cm^3^)	Density *ρ*(+) (g/cm^3^)	Density *ρ*(+) (g/cm^3^)
200	10	20	5
100	5	10	2
50	2	6	1
10	2	4	0.5
5	2	4	0.5

## Data Availability

Interactive graphic surfaces are provided on a web-based platform to help in the selection of the best combination of restorative crown materials. All files are freely ac-cessible on a Git repository page under the MIT Open software license (https://xaviermarimon.github.io/CrownMaterials).

## Abbreviations

MET	CoCr Metal
MCOM	CoCr Metal-Composite
MCER	CoCr Metal-Ceramic
FCOM	Carbon Fiber-Composite
PKCOM	PEEK-Composite
FCCER	Carbon Fiber-Ceramic
